# A Systematic Review to Identify the Effects of Biologics in the Feet of Patients with Rheumatoid Arthritis

**DOI:** 10.3390/medicina57010023

**Published:** 2020-12-29

**Authors:** Laura Ramos-Petersen, Christopher James Nester, Andres Reinoso-Cobo, Pilar Nieto-Gil, Ana Belen Ortega-Avila, Gabriel Gijon-Nogueron

**Affiliations:** 1 Department Podiatry, Universidad Católica San Antonio de Murcia, 30107 Murcia, Spain; lrpetersen@ucam.edu; 2 School of Health & Society, University of Salford, Salford M5 4WT, UK; C.J.Nester@salford.ac.uk; 3 Department Podiatry, University of Malaga, Arquitecto Francisco Penalosa 3, 29071 Malaga, Spain; andreicob@uma.es (A.R.-C.); gagijon@uma.es (G.G.-N.); 4 Department of Nursing and Podiatry, University of Valencia, 46010 Valencia, Spain; pilar.nieto@uv.es; 5 Instituto de Investigación Biomedica de Malaga (IBIMA), 29071 Malaga, Spain

**Keywords:** biologics, DMARDs, feet, rheumatoid arthritis, systematic review

## Abstract

*Background and Objective:* Ninety percent of patients with rheumatoid arthritis (RA) feel foot pain during the disease process. Pharmacological treatment of RA has a systematic effect on the body and includes: Nonsteroidal anti-inflammatory drugs, disease-modifying antirheumatic drugs (DMARDs) and biologics. The objective of our review was to examine the impact of biologics on patients with RA ‘foot. *Methods and Material:* A systematic review of randomized control trials and observational studies that evaluated the efficacy of biologics against other pharmacological treatment, and included a foot outcome measure. The search covered MEDLINE Ovid, Pubmed, CINAHL, Cochrane Library, Evidence Search, and Web of Science. Risk of bias was evaluated using Cochrane guidance and the Newcastle Ottawa Scale adapted version. *Results:* A total of eight studies fully met the inclusion criteria: Three randomized control trials, and five observational studies were the basis of our review. A total sample of 1856 RA patients with RA treatment participated. The use of biologics was not associated as a risk factor for post-operative surgical site infection or delayed wound healing. The benefits of biologics, in terms of the disease evolution, were assessed using X-ray. *Conclusion:* Evidence suggests that the use of biologics is not a risk factor for post-operative surgical site infection or delayed wound healing. The use of biologics presents benefits in terms of the disease evolution assessed through X-ray.

## 1. Introduction

Rheumatoid arthritis (RA) is a musculoskeletal disorder with a chronic inflammatory autoimmune condition that commonly affects foot joints, ankles, knees, and wrists [1]. It impairs normal daily life, affects body image and personal relationships, and therefore, also impacts the quality of life [2,3,4]. There is, consequently, a significant social and economic cost [4]. Foot involvement and foot joint pain are signature features of early RA and almost omnipresent during the progress of the disease, with subsequent physical and psychosocial impairment [5,6]. The prevalence of foot pain increases with disease duration, affecting 90% of people with RA at some stage [7,8]. The current strategy, as defined by the RA guidelines, is a treat-to-target strategy. The purpose of this strategy is to treat active RA to achieve a target of remission or lower disease activity in cases that remission cannot be achieved. The RA guidelines also state that it is important to always consider people’s rights to be involved in discussions and make informed decisions about their care. The purpose is to provide pain relief, preserve physical activity, and quality of life [9,10]. Treatments include pharmacological agents, but also footwear, foot orthoses, and sometimes surgery [11].

Nonsteroidal anti-inflammatory drugs (NSAIDs) are commonly used, but do not modify the disease evolution. In contrast, disease-modifying antirheumatic drugs (DMARDs) have the capacity to slow disease progression (including methotrexate (MTX), sulfasalazine (SSZ), and leflunomide [12]). These can alleviate patient symptoms, and if used early in the RA process, can enable better longer-term outcomes [13]. Biologics are a special type of DMARD, and they can help in terms of limiting radiological damage by inhibiting joint destruction and suppressing inflammation [14,15,16]. However, some patients do not respond to pharmacological treatments, or initial responses may reduce and efficacy changes over time. Regardless, any treatment has the capacity as an anti-RA strategy if it inhibits hyperplasia of synovial cells [17,18]. A previous systematic review concludes that compared with DMARDs alone, biologics, in combination with DMARDs achieve a 50% reduction of joint destruction [19]. Regarding to biologics, they have shown significant contribution in aiding the reduction of inflammation and articular destruction [20], which may indicate clinical benefits in terms of the feet of RA patients. A systematic review of the influence of biologics effects on RA patients in general pain, concluded that biologics are clearly effective in pain relief, improving functional status, and preventing structural joint damage [21]. A prior qualitative study about foot impairments in RA patients with biologics described various participation limitations related to foot problems, such as foot impairments influencing work or foot obstacles in domestic life, [22] without any foot pain mentioned. However, the effect of biological treatments on the foot is not well-known, since there are not many randomized control trials (RCTs).

A recent meta-analysis of evidence related to footwear and orthoses has evidenced their efficacy in relation to the reduction of foot pain and associated disability and increased quality of life [23]. A similar appraisal of the literature concerning pharmacological treatments has not been published. The objective of our systematic review was to explore biologics effects in patients with rheumatoid arthritis in terms of their feet.

## 2. Materials and Methods

Review registration number: PROSPERO CRD42019137893.

This review was performed following the Preferred Reporting Items for Systematic Reviews and Meta-Analyses (PRISMA) statement [24].

### 2.1. Search

Studies were analyzed under the PRISMA guidelines [24], and a search was carried out from inception using the following databases: MEDLINE Ovid, Pubmed, CINAHL, Cochrane Library, Evidence Search, and Web of Science. A previous scoping search was carried out to ensure that this aim had not been addressed by previous studies, and PROSPERO and Cochrane Library were explored. The last search was run on 9th April 2020 by one reviewer. The following Mesh terms were used to identify relevant clinical trials: “Arthritis” [MeSH Terms], “Rheumatoid” [MeSH Terms], “Foot” [MeSH Terms], “biologics” [MeSH Terms], “biological therapy” [MeSH Terms] (Appendix A).

Eligibility criteria, study selection, and data collection process:

We reviewed studies that assessed the efficacy of biologics therapy in terms of RA patients’ feet. All studies conducted the following PICO structure [25]:

− P (population) = female and male patients with RA, aged > 18 years.
− I (intervention) = efficacy of biologics treatment in terms of RA patients’ feet.
− C (comparator) = other type of pharmacological or conservative treatments.
− O (outcome) = evaluation of biologics effects on RA patients ´feet, as the modified Sharp-van der Heijde (SvdH) score [26] or the use of the Guideline for the Prevention of Surgical Site Infection [27] or any aspect that directly affects the measurement of the foot.

No publication status restrictions or publication date were imposed. Randomized control trials (RCT) and observational studies were included.

Studies focused on animals, lupus, juvenile or psoriasis arthritis were excluded. Studies that did not include biologics therapy or it was not compared with other pharmacological or conservative treatments, systematic reviews, non-focused on RA patients ´feet, case reports, skin cancer, or studies in other languages rather than English were also excluded. 

### 2.2. Study Selection

Study selection was carried out independently by two reviewers in an unblinded, standardized manner. They extracted data from included studies, and disagreements between them were resolved by consensus.

### 2.3. Data Extraction and Analysis

Two reviewers independently screened titles of potentially included studies to identify studies that may have met the inclusion criteria outlined above. Then, the studies were screened via their abstract. Finally, full texts of possibly eligible studies were investigated. Any disagreement between reviewers over the eligibility of studies was discussed with a third reviewer. The data extracted was study details (author, country, and year of publication), sample size (gender, years of age, number of participants with), blinding, follow-up, intervention, measurement instrument used, and results.

Whilst it was an aspiration at the start, due to the heterogeneity of studies and the varying outcomes, a meta-analysis was not appropriate.

### 2.4. Risk of Bias in Individual Studies

Two reviewers worked unblinded to evaluate the risk of bias in individual studies, using the Cochrane Handbook for Systematic Reviews of Interventions (CHSRI) [25] to evaluate randomized control trials (RCT) and the Newcastle-Ottawa Scale (NOS) [28] for observational studies. NOS is a reliable and valid tool to evaluate the quality of any observational design that has an adapted version that has been used by previous systematic reviews [29]. The NOS adapted version assesses the risk of bias, including four domains: Selection bias, performance bias, detection bias, and information bias. Those domains contain seven items, each item is scored from zero (high risk) to three (low risk) points. Therefore, a study is considered a high risk of bias with a total score from 0 to 6, moderate risk of bias from 7 to 13, and a low risk of bias from 14 to 21. 

Reviewers assessed each RCT taking in account the following domains from the (CHSRI): Bias arising from the randomization process; bias due to deviations from intended interventions; bias due to missing outcome data; bias in the measurement of the outcome and bias in the selection of the reported result. Allocation, blinding, incomplete outcome data, selective reporting, and other potential sources of bias are included in the table. 

We used the Review Manager (RevMan) (Computer program). Version 5.3. Copenhagen: The Nordic Cochrane Centre, The Cochrane Collaboration, 2014.

## 3. Results

**Figure 1:**
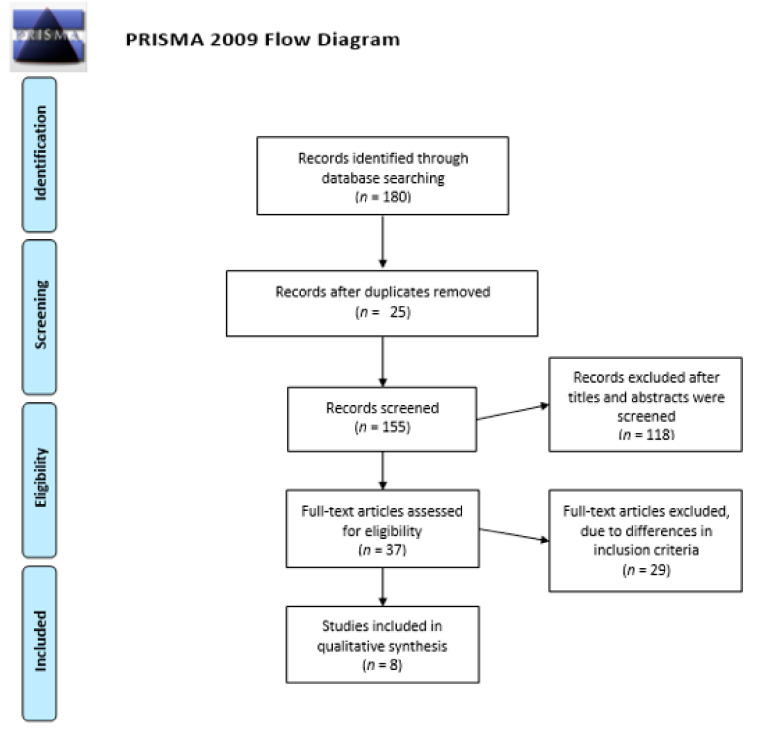
PRISMA flow diagram.

Searches identified 180 articles, reduced to 155 after duplications were removed. These were screened by title and abstract, and 118 were excluded. The remaining 37 were assessed, and 8 carried forward. Twenty-nine studies were excluded, due to differences in inclusion criteria, as no additional treatment for comparison or use of non-humans, meaning comparison of data would not be possible. Thus, only eight studies fully met the inclusion criteria and were the basis of our review. Three randomized control trials and five observational studies (four retrospective studies and one prospective study). The PRISMA flow diagram is described in Figure 1.

### 3.1. Study Characteristics and Syntheses of Results

They were published in English and between 2004 and 2016. The duration of the intervention was between 12 months and over five years. The total number of participants involved was 1856. Five of the eight studies, include information about gender, showing that 965 participants (51.5%) were female. One of the RCT had blinded participants (Table 1).

All the studies included foot related outcomes, as radiographic disease progression, surgical site infection (SSI), development of infection, or wound healing. General health status-related, or RA assessment were also measured using the Health-Assessment Questionnaire (HAQ); the Disease Activity Score (DAS28), and the EuroQol-5D (EQ5D), which includes daily functioning, quality of life, radiographic progression, and adverse events. However, this systematic review is focused on foot outcomes, therefore, this information was not included within our review.

### 3.2. Surgical Site Infection

SSI was assessed in five (62.5%) of the included studies. SSI outcomes were diagnosed based on the Guideline for the Prevention of Surgical Site Infection [27]. By mutual agreement in all the studies, the use of biologics is not a risk factor for post-operative SSI [30,31,34,36,37].

### 3.3. Delayed Wound Healing

That outcome was quantified in three of the eight included studies. Delayed wound healing was defined as either delayed suture removal or exhibit impaired healing, and this was judged by physicians [27,38]. All the studies concluded that biologics use is not a risk factor for delayed wound healing [30,31,36].

### 3.4. Radiographic Progression

Three studies included outcomes to measure the disease evolution assessed through X-ray to know biologics effects in terms of patients´ feet. Radiographs were assessed using the modified Sharp-van der Heijde (SvdH) score (range 0–448; higher scores indicate more joint damage). These values included subscores for erosion (range 0–280) and joint space narrowing (range 0–168) [26]. Also, radiographs were assessed by applying the Genant-modified Sharp Score (GSS). Two different results were found in the included studies: Relevant radiographic progression differences were not found between the groups [32,33] and less radiological disease progression was found in patients with biologics [35].

### 3.5. Risk of Bias

**Figure 2:**
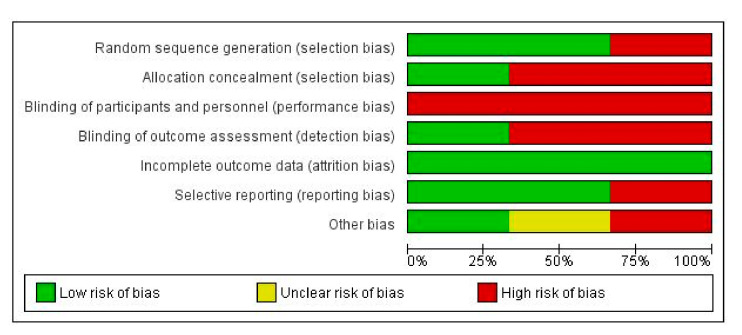
Risk of bias graph.

**Figure 3:**
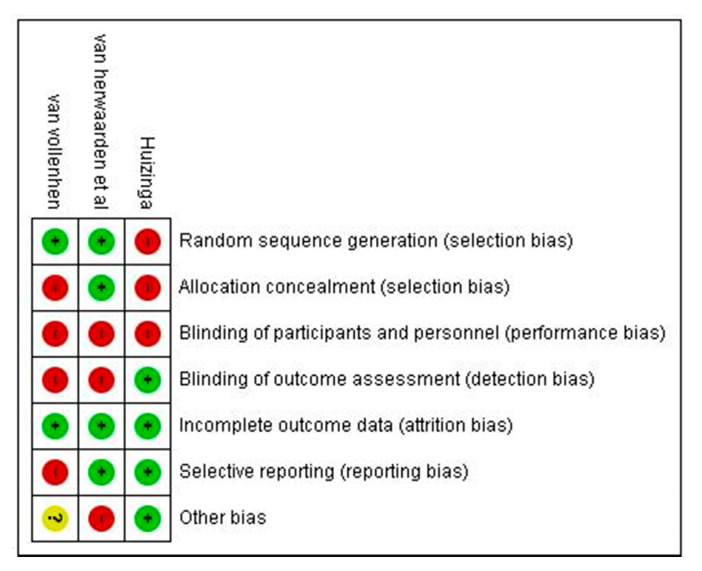
Risk of bias summary.

Risk of bias was evaluated using Cochrane guidance within RCT included studies is Figure 2 and Figure 3. All RCTs had low quality in the blinding of participants and personnel, and most RCTs had uncertainty in allocation concealment and blinding of outcome assessment. The risk of bias assessment of observational studies is presented in Table 2, showing one moderate risk of bias study and four low risks of bias studies by NOS adapted version.

## 4. Discussion

The main aim of our systematic review was to evaluate the evidence for changes in foot outcomes in patients with RA using biologics. From the review above, key findings emerge: Longitudinal analyses reported that the use of biologics may not be a risk factor for post-operative surgical site infection or delayed wound healing, and there are no differences between biologics and non-biologics in terms of radiographic progression. Those are some important findings in the understanding of the biologics effect on RA patients´ feet.

Our initial hypothesis was that the use of biologics could benefit patients with RA in terms of their feet, such as reducing foot pain, and therefore, improving quality of life. Most patients with RA report foot symptoms during the process of the disease, foot pain being the most common [6]. Whilst based on suitable studies, our analysis of eight studies involving almost 2000 participants did not report any changes in terms of foot pain. Their outcomes are related to feet, including radiographic disease progression, SSI, development of infectious or wound healing. The included studies evaluated pain and quality of life from a holistic patient perspective. Those studies concluded that biologics can be used to improve patients’ pain, but there was no specific indication of foot pain. A previous qualitative study explored the personal experiences of patients with RA in receipt of biologics, in terms of their feet. In this qualitative research, patients described that before biologics, they felt more pain and disabling symptoms. Also, patients declared that their function and mobility were restored. However, patients reported that foot pain remained, which could be explained by the established deformity or foot surgeries [39]. There is a lack of experimental studies focused on foot pain outcomes in RA patients receiving biologics. 

Regarding the gender influence in our included studies, this information could not be found in all of them. Only five studies provide data related to sex difference, showing that most of the overall participants were female [30,32,33,35,37]. This fact agrees with the previous findings of RA, which identifies that the disease is more common in women than men (3:1) [40]. A prior review about gender influence in RA demonstrated that males and females approach their pathology differently. The presence of comorbidities, such as fibromyalgia, a different immune response, major depression, hormonal differences, and osteoporosis, are more frequent in females [41]. It may influence the results; therefore, it is necessary to distinguish results from females and males in future research.

It has been suggested that patients with biologics have an extensive risk of post-operative infection [42], and the British Society for Rheumatology (BSR) have developed guidelines for the management of the biologic agent Tocilizumab. BSR claims that it is necessary to balance the risks of post-operative infection against the risks of a post-operative disease flare [43]. Some studies are investigating the risks associated with discontinuation of biologics vs. the resultant infection risk after surgery. Our findings on post-operative infection suggest that the use of biological is not a risk factor for post-operative infection in foot and ankle surgery. Tada et al. in 2016 [30], in their retrospective study among 227 patients with RA after 332 elective orthopedic surgeries, concluded that biologics were not risk factors for post-operative SSI. They concluded that the risk factor for post-operative SSI was foot surgery, due to the severe foot deformities, which causes swelling and increased skin turgor. Kadota et al. [31] also found similar results, which provides support for the present findings. Therefore, surgeons and healthcare professionals who are involved in wound care need to be aware that foot surgery may be associated with SSI complications.

Previous studies reported that there is conflict related to continuing or stopping biologic drug therapy prior to orthopedic procedures in terms of avoiding the possible side effects of these drugs in delayed wound healing [44]. Previous in vivo studies, focused on the overall impact of biologics upon wound healing, showed that biologics suppress the promotion of key structural proteins, but help to collagen synthesis [45,46]. Nevertheless, a real environment is not considered in vivo studies. Included studies, within a real orthopedic surgery process, concluded that biologics use is not a risk factor for delayed wound healing [30,31,36]. 

The assessment to determine bias within the observational studies, which we included, demonstrated four low risk, and one moderate risk study. The studies also contained missing data, such as activity remission, and the authors also showed a conflict of interest. The risk of bias within the randomized control trials included in this review, presented incomplete outcome data and a lack of blinding. There was a large difference between the number of included participants in the studies, the lowest of which being 31 [37], and the highest being 556 [33]. One of the strengths of this review is the use of specific review tools and checklists to evaluate the risk of bias.

The clinical implications of our results may help in the therapy election for patients with RA in terms of their feet, considering the benefits in the feet radiographic progression. The applicability of these new results is also shown in the perioperative process. 

The main limitation is that we have focused our review on biologics on foot outcomes instead of the effectivity of all therapies for RA on patients’ foot. Due to the nature of the study, groups were very heterogeneous, and sometimes information, such as the types of surgery performed or if biologics were stopped peri-operatively, which may have an impact on SSI following surgery, were not clear. Another limitation of the present study is the difficulty related to finding papers that relate to the topic of our study. This is due to the ambiguity in the studies related to RA, which mention feet, however, the aim of the study is not foot related. This makes it impossible to analyze the repercussion of biologics in terms of feet patients. 

## 5. Conclusions

The included studies suggest that the use of biologics slows the rate of foot joint erosion. Our review shows that biologics are not a risk for surgical site infection or delayed wound healing after foot and ankle surgery. However, as the included studies do not define if the use of biologics stopped prior to the surgery, results should be taken into consideration with caution. More research focused on biologics effect on foot pain is needed. We strongly suggest that biologics continue to be studied in experimental settings for the treatment of foot pathology in RA patients. Due to the diversity within the methodology of the included studies, results should be taken into consideration with caution. Thus, more rigorous and larger studies are needed.

## Figures and Tables

**Table 1:** Study characteristics.

Author and Year	Country/Study Type	Sample Size	Blinding	Follow-Up	Intervention	Foot Outcome	Results
**Tada et al., 2016** [30]	Japan Retrospective study	227 patients with RA Mean of age 65.0 *n* female = 197 (86.7%) Rates of biologics and conventional synthetic DMARD (csDMARD) administration were 30.4 and 91.0%, respectively.	No	Between 2006 and 2013	Orthopedic surgeries. Disease Activity Score (DAS28)	Surgical site infection (SSI). (odds ratio (OR), 1.11; *p* = 0.045), Wound healing (OR, 3.66; *p* = 0.003).	Biologics were not risk factors for post-operative SSI. Foot surgery was a risk factor for delayed wound healing, due to the severe foot deformities, which causes swelling and increased skin turgor
**Kadota et al., 2016** [31]	Japan Retrospective study	204 foot and ankle surgeries in RA patients. 157 with biologics treatment and 47 with csDMARD treatment.	No	Between January 2004 and December 2012	Orthopedic procedures.	SSI (OR), 3.167; Confidential Interval (CI), 1.256–7.986; *p* = 0.015). Delayed wound healing (DWH) (OR 1.004; CI, 1.000–1.007; *p* = 0.029)	SSI and DWH were identified in 8 cases (7 with csDMARD treatment) and 3 cases (2 with csDMARD treatment), respectively. Foot and ankle surgery were associated with an increased risk of SSI.
**Van Herwaarden et al., 2015** [32]	Netherlands Randomize Control Trial (RCT)	180 patients with RA: *n* = 121 with biologics and dose reduction Mean of age 59 *n* female = 75 (61%) *n* = 59 without dose reduction (usual care). Mean of age 58 *n* female = 41 (69%)	No	18 months	Biologics vs. usual care in RA DAS28 Health assessment questionnaire–disability index (HAQ–DI). EuroQol-5D Cumulative incidence of flares	Radiological outcomes short lived flares (73% v 27%) and minimal radiographic progression (32% v 15%)	Biologics are non-inferior to usual care regarding outcomes
**Huizinga et al., 2015** [33]	Multicentre: Netherlands, UK, Spain, Germany, Israel, Brazil USA, Switzerland and France.RCT	From 556 randomized patients, *N* = 279 biologics tocilizumab (TCZ) + methotrexate (MTX) (add-on) and *n* females = 227 (81.9%) Mean of age 53 *n* = 277 TCZ + (Placebo)PBO (switch). *n* females = 217 (78.6%) Mean of age 53.6 Completed week 104: *n* = 222 TCZ + MTX (add-on) *n* = 201 TCZ + PBO (switch).	Yes	Over 24 months	Patients with active RA despite MTX were randomized to add TCZ to ongoing MTX (add-on) or switch to TCZ plus placebo (PBO) (switch). Disease Activity Score (DAS28) RA quality of life questionnaire Tender joint count. Swollen joint count. HAQ–DI. Patient’s global assessment. Physician’s global assessment. C-reactive protein	Radiographs of hands/wrists and feet. 50.4% discontinued TCZ after achieving sustained remission, and 5.9% achieved drug-free remission	Most patients demonstrated minimal progression of radiographic structural damage, with differences favoring the add-on group (*p* = 0.034). Serious adverse events and serious infections per 100 patient-years were 12.2 and 4.4 in add-on and 15.0 and 3.7 in switch patients.
**Kubota et al., 2014** [34]	Japan Retrospective study	87 foot and ankle surgeries in RA patients. 50 with biologics and 37 with non-biologics.	No	Between January 2006 and December 2011.	Orthopedic surgery.	SSI (*p* = 0.001), (OR)19.27; (CI) 4.67–79.45]. Late infection	The use of biologics does not significantly increase the incidences of SSI and late infection after orthopedic surgery
**Van Vollenhoven et al., 2012** [35]	Sweden RCT	487 patients with RA and previous treatment with MTX. After 3–4 months, those who their treatment failed:*n* = 130 (group A) with conventional treatment Mean of age 52.9 *n* female = 101 (78%) *n* = 128 (group B) with biologics. Mean of age 51.1 *n* female= 79 (76%)	No	24 months	Addition of conventional disease modifying antirheumatic drugs (group A) vs. addition of biologics (group B) vs. DAS28 HAQ-DI Health-economic outcomes	Radiological outcomes (mean 7·23 Standard deviation (SD) 12·72) vs. 4·00 (10·0); *p* = 0·009).	In group B, good response was non-significantly greater than it was in group A. After 24 months, radiological disease progression was greater in patients in group A than it was in those in group B (*p* = 0·009).
**Kubota et al., 2012** [36]	Japan Retrospective study	84 foot and ankle surgeries in RA patients. 47 with biologics and 37 with non-biologics	No	Between January 2006 and December 2010	Orthopedic surgery.	SSI (*p* = 0.956) Late infection (*p* = 0.55)	No statistically significant difference between groups. The use of biologics may not affect the incidence of post-operative wound healing and SSI.
**Bibbo et al., 2004** [37]	USA Prospective study	*n* = 28 females (90%) overall *n* = 16 biologics (group 1) *n* = 15 not receive biologics (group 2)	No	12 months	Risk for healing and infectious complications smoking history	Development of infectious/healing complication (*p* = 0.033).	Group 1 demonstrated a lower complication rate (*p* = 0.033) in healing and infection.

**Table 2:** Risk of bias assessment for observational studies (NOS adapted version).

Study	Selection Bias	Performance Bias		Detection Bias		Information Bias		Total Score
	A	B	C	D	E	F	G	
Tada et al. [30]	3	2	0	3	3	3	3	17
Kadota et al. [31]	3	1	1	3	3	2	3	16
Kubota et al. [34]	2	1	2	2	3	2	3	15
Kubota et al. [36]	2	1	2	2	3	2	3	15
Bibbo et al. [37]	1	2	0	0	3	1	2	9

Note: A = Is the source population appropriate and representative of the population of interest?; B = Is the sample size adequate, and is there sufficient power to detect a meaningful difference in the outcome of interest?; C = Did the study identify and adjust for any variables or confounders that may influence the outcome?; D = Did the study use appropriate statistical analysis methods relative to the outcome of interest?; E = Is there little missing data, and did the study handle it accordingly?; F = Is the methodology of the outcome measurement explicitly stated, and is it appropriate?; G = Is there an objective assessment of the outcome of interest?

## Data Availability

Data sharing not applicable.

## Appendix A. Search Strategies

Database: Ovid MEDLINE(R) and Epub Ahead of Print, In-Process & Other Non-Indexed Citations, Daily and Versions(R) (1946 to April 08, 2020)

Search Strategy:

1. exp Arthritis, Rheumatoid/ (111898)
2. exp Foot Joints/or exp Foot/ (62425)
3. exp Biological Therapy/ (663983)
4. 1 and 2 and 3 (18)

Database CINAHL:

rheumatoid arthritis AND foot AND (biologics OR biological therapy) (33)

Database Cochrane Library:

rheumatoid arthritis AND foot AND (biologics OR biological therapy) in Title Abstract Keyword (23)

Database Evidence Search:

rheumatoid arthritis AND foot AND (biologics OR biological therapy) (40)

Database Pubmed:

(((rheumatoid arthritis) AND (foot OR feet)) AND (biologics)) AND (biological therapy) (67)

Database Web of Science:

((rheumatoid) arthritis AND foot) AND (biologics) OR biological therapy)) (18)
